# The Signposting Questionnaire for Autism-2 (SQ-A-2): using input from autistic adults to inform questionnaire development

**DOI:** 10.1186/s13229-026-00714-3

**Published:** 2026-04-15

**Authors:** Lucy A. Livingston, Susan R. Leekam, Gareth Davies, Punit Shah, Olujolagbe Layinka, Sarah J. Carrington, Catherine R. G. Jones

**Affiliations:** 1 Department of Psychology, Institute of Psychiatry, Psychology and Neuroscience, King’s College London, London, UK; 2 Wales Autism Research Centre, School of Psychology, Cardiff University, 70 Park Place, Cardiff, CF10 3AT UK; 3 Neuroscience and Mental Health Innovation Institute, Cardiff University, Cardiff, UK; 4 South Wales Doctoral Programme in Clinical Psychology, Cardiff University, Cardiff, UK; 5 Department of Psychology, University of Bath, Bath, UK; 6 Department of Psychology, Aston University, Birmingham, UK

**Keywords:** Autism, Diagnosis, Adults, Signposting, Autistic traits

## Abstract

**Background:**

Brief questionnaires help clinicians to identify people who may require in-depth assessment for Autism Spectrum Disorder (hereafter autism) and also facilitate research. However, many current questionnaires do not map onto the latest DSM-5 criteria for autism and were developed by researchers and/or clinicians, with limited input from autistic people. The Signposting Questionnaire for Autism (SQ-A) is a caregiver-report questionnaire for measuring autism signs in children, based on DSM-5 criteria. Here, we developed a revised lifespan version, the SQ-A-2, with input on wording from autistic adults. We tested the SQ-A-2 as a self-report measure in autistic and non-autistic adults, and assessed the impact of the adapted wording on both the psychometric properties and the acceptability of the measure.

**Methods:**

The SQ-A, containing highly discriminating DSM-5 items from a valid clinical interview, was revised to form the 18-item SQ-A-2, with self- and informant-report options. Following input from a team of four autistic consultants and six autism professionals, item wording on the SQ-A-2 was adapted, thus forming Original and Adapted versions. Subsequently, a sample of autistic and non-autistic adults (N = 302), matched on age, sex, and general cognitive ability, completed both versions of the new SQ-A-2, as well as the widely-used 10-item Autism-Quotient (AQ-10). Autistic participants also rated their preferences for Original versus Adapted items, and provided qualitative responses for their reasoning.

**Results:**

Overall, the self-report version of the SQ-A-2 demonstrated excellent internal reliability and convergent validity with the AQ-10, and autistic adults scored significantly higher than non-autistic adults. This group difference was greater for the Adapted compared to the Original version of the SQ-A-2, suggesting autistic consultation may facilitate the development of clinically-useful measures. Additionally, autistic participants preferred the wording of the Adapted versus Original items, both quantitatively and qualitatively.

**Limitations:**

Our findings may not generalise to autistic people with intellectual disability. Ethnicity data was not collected.

**Conclusions:**

The adapted version of the SQ-A-2 has the potential to be a valid, clinically useful questionnaire for measuring DSM-5 signs of autism across the lifespan. Our findings also highlight the utility of incorporating autistic input into measure development. Further psychometric testing of the SQ-A-2 in larger, more diverse samples, including the informant-report version, is now required.

**Supplementary Information:**

The online version contains supplementary material available at 10.1186/s13229-026-00714-3.

## Background

The number of people being diagnosed with Autism Spectrum Disorder (ASD; hereafter autism) has been rapidly increasing worldwide [1], reflected in a rising demand for diagnostic assessments. Brief, self- or informant-report questionnaires provide an efficient and cost-effective method to support and streamline the diagnostic process. They can be used by frontline professionals, including those who do not have specialist autism knowledge, to refer people for an in-depth autism assessment. They can also supplement the diagnostic assessment itself alongside more complex behavioural (e.g., Autism Diagnostic Observation Schedule [2] and interview-based (e.g., Diagnostic Interview for Social and Communication Disorders, DISCO [3, 4] methods. Additionally, with considerable waiting times after initial referral for an assessment [5], such questionnaires can also give insight into a person’s autistic profile and thus their support needs, whilst they await assessment.

There is a particular need for autism questionnaires that are suitable for adults. In recent years, the sharpest increases in autism diagnoses have been seen amongst adults; for example, in England, there was a > 1300% increase in adult diagnoses from 1998 to 2018 [6], with a similar trend found in Wales from 2001 to 2016 [7]. Internationally, the age-standardised prevalence of autism has increased over the past three decades in countries with high socio-demographic indices [8]. Notably, some countries with low socio-demographic indices have seen a decrease in prevalence, which has been related to issues around awareness and limited access to screening tools and diagnostic services [8]. Increase in autism diagnosis has been attributed to greater public and clinical awareness that autism is lifelong and may go missed in earlier life [9, 10], as well as greater access to specific adult diagnostic services (e.g., following the ASD Strategic Action Plan [11, 12] in Wales, the Autism Act [13] in England, the National Autism Strategy [14] in Australia, the Autism Innovation Strategy [15] in Ireland). Whilst there are numerous lengthy questionnaires to measure autistic traits in the adult general population—including various iterations of the Autism-Spectrum Quotient (AQ [16–18], the Broad Autism Phenotype Questionnaire [19], and the Comprehensive Autistic Trait Inventory [20]—questionnaires suitable for clinical practice are limited. Globally, there is variance in guidance for autism diagnostic assessment and the evidence base for brief signposting measures is limited [21–23]. As such, most countries do not formally recommend a specific brief questionnaire to support the autism adult diagnostic process. However, since 2012, the UK’s National Institute for Health and Care Excellence [24] has recommended that adults without intellectual disability who score 6 or above out of 10 on the 10-item Autism-Spectrum Quotient (AQ-10 [18] should be considered for a full diagnostic assessment (although see [25, 26]). As such, the AQ-10 is the most widely used brief questionnaire in the UK. Its popularity has been underpinned by its brevity, along with high specificity and sensitivity for classifying autism [18] and comparable performance to the full 50-item AQ [27]. An additional self-report tool, designed specifically for use with autistic adults in clinical practice but less widely adopted in the UK, is the 14-item Ritvo Autism Asperger Diagnostic Scale (RAADS-14 [28].

There are, however, concerns that the currently available autism questionnaires, for both children and adults, may be limited. Beyond ongoing psychometric issues [29–31], many measures were not born from clinical diagnostic assessments and therefore do not optimally map onto the latest diagnostic criteria, as specified by the DSM-5 [32] and ICD-11 [33]. The AQ-10 [18], which represents the 10 most discriminating items from the full 50-item measure developed for research purposes [17], contains certain items (e.g., on attention switching) that do not reflect core diagnostic features of autism. Similarly, whilst the presence of sensory sensitivities was added to the diagnostic criteria in DSM-5 [32], this tends not to be well-represented in current measures. For example, the AQ-10 only includes one sensory item and this is specific to auditory hypersensitivity, whilst the full AQ [17] is more focused on the cognitive construct of attention to detail rather than related behavioural constructs of sensory processing and sensory pleasure or distress. As such, it is unsurprising that these measures may not necessarily predict who will go onto receive a diagnosis [34, 35], do not always correlate with gold-standarddiagnostic tools [34], and may not optimally differentiate autism from closely-related neurodevelopmental (e.g., ADHD) and mental health (e.g.Generalised Anxiety Disorder) conditions in clinical settings [34] (see [36] for detailed discussion).

A final concern, often raised by autistic people and their families, is that widely-used autism questionnaires may not always capture autistic people’s perceptions of their autistic characteristics. Such questionnaires have been critiqued, for example, for being biased towards particular sub-groups of people (e.g., males) and containing overly clinical and/or deficit-focused language [37]. Indeed, only a handful of autism (e.g. [20, 37] and autism-relevant (e.g. [38–40] questionnaires have involved autistic consultation in their development, typically with a small group of people. It is not clear how representative these views are of the much wider autistic population, and no research, to our knowledge, has assessed how consultation might impact item endorsement and more general measurement properties. There are non-trivial challenges associated with developing measurement tools that represent and respect autistic people, whilst still serving clinical utility in guiding timely and accurate diagnoses. For example, even a small difference in language or its framing could potentially shift autistic peoples’ response on a Likert scale. Equally, such changes might also alter non-autistic peoples’ responses, with critical consequences for the measure’s validity, sensitivity, specificity and thus clinical utility.

### Signposting Questionnaire for Autism (SQ-A)

The 14-item Signposting Questionnaire for Autism (SQ-A [41] was developed as a brief, caregiver-report measure of autism signs. Crucially, it was derived from the 14 most discriminating items of the DISCO [3, 4], a widely-used diagnostic clinical interview with an established DSM-5 algorithm [42]. These 14 DISCO interview items were originally identified based on their predictive validity for discriminating confirmed clinical diagnoses of autism from confirmed diagnoses of intellectual disability or language impairment [43, 44]. When developed into a questionnaire, the SQ-A demonstrated good psychometric properties, including construct validity and internal consistency (α = 0.87), as well as specificity in differentiating autistic children from those with other neurodevelopmental conditions [41].

The SQ-A is an example of an autism questionnaire that balances brevity, as is often required in healthcare settings, with clinical utility. Although four response options per item are presented to respondents, the scoring system reduces this to a binary scoring system according to syntax rules reported by Carrington et al. [44] and Jones et al. [41]. This adaptation streamlines the scoring and makes the total score more readily interpretable. It also holds promise as a lifespan autism measure, which with further testing could be suitable for use in children and adults. The DISCO was designed to facilitate assessment of multiple domains of development and functioning at any age. Additionally it can be used to facilitate autism diagnosis when using the algorithms items within the DISCO; with parent-report being used for child diagnoses, and adults either being interviewed themselves, or where appropriate, an informant such as a parent being interviewed instead (e.g., if a person has intellectual disability and/or language impairment). Therefore, the SQ-A items are inherently relevant across the lifespan. There is considerable benefit to brief autism questionnaires having utility across the lifespan, for both research and clinical practice. Not only does this enable autistic characteristics to be more readily tracked across different ages, both within and between people, but this may be particularly useful during transition ages (e.g., from adolescence to adulthood) when changes may indicate the need for greater understanding and support. Additionally, developing a measure with the potential for use across the lifespan and with both self- and informant-report options means that it can be inclusive of a wide range of the autistic population, including those with additional intellectual disability, language impairment and/or support needs, who might otherwise not be able to self-report (e.g., using the AQ-10). The SQ-A-2, as developed in the current research, is designed to be suitable across the lifespan with both self- and informant-report versions, although the current study focuses on the self-report version in adults.

### Current study

Building on the original SQ-A work in children [41], we aimed to develop a revised version, the SQ-A-2, as a brief measure of autism signs that will be tested on adults. The SQ-A-2 includes additional items from the DISCO, and items were adapted in language/phrasing following consultation with four autistic consultants. In the current study, a large sample of autistic and non-autistic adults (*N* = 302) completed two versions of the SQ-A-2: the Original version (pre-consultation), which uses the same wording as the SQ-A, and the Adapted version (post-consultation). Further, autistic participants provided quantitative and qualitative responses on their preferences for Original versus Adapted items.

Overall, we addressed two research aims. First, we tested the SQ-A-2 questionnaire in adults for the first time, assessing its internal reliability, convergent validity, and discrimination between autistic and non-autistic adults. We assessed convergent validity by comparing the SQ-A-2 to the AQ-10 [18], chosen as it is the most widely used brief measure of autistic features. Second, we assessed the impact of adapting questionnaire items based on autistic input. Specifically, we tested whether this influenced item endorsement and measurement properties of the questionnaire; a novel analysis which has, to our knowledge, never been assessed for previous autism measures. Additionally, by collecting preference data from over 150 autistic participants, we simultaneously assessed whether the language changes to the SQ-A-2, informed by a small group of autistic adults, were supported by a much larger sample of the autistic population.

## Methods

### Participants

Participants were 302 adults in the UK (146 females), aged 18–67 years (*M* = 31.10, *SD* = 10.12), recruited via the online research platform *Prolific*. This included 151 participants with a self-reported clinical autism diagnosis and 151 non-autistic participants, matched on age and sex. The matching process involved first recruiting non-autistic participants with a roughly equal split of males and females, and subsequently recruiting autistic participants who matched the non-autistic participants across sex and age bands. Autistic participants self-reported their diagnosis type (e.g., Asperger Syndrome; Autism Spectrum Disorder) and the profession(s) of the people who made the diagnosis (e.g., Clinical Psychologist, Psychiatrist). Non-autistic participants confirmed that they did not have a clinical autism diagnosis or suspect they were autistic. Autistic and Non-autistic groups did not significantly differ in terms of age (*p* =.31, *d* = 0.12) or general cognitive ability (*p* =.31, *d* = 0.12) and were equally balanced with 78 males and 73 females each. Full participant characteristics are shown in Table 1. Power analysis showed we had 91% power in the group-comparisons, and 99% in the within-autism group analyses to detect small-to-medium effect sizes (*d* = 0.35; *α* = 0.05; two-tailed).

**Table 1:** Autistic and non-autistic group characteristics

	Autistic	Non-autistic
	(n = 151)	(n = 151)
	M(SD), range	M(SD), range
Age	30.51(9.59), 18-63	31.70(10.62), 18-67
General Cognitive Ability _max = 16_	8.46(3.68), 0-16	8.05(3.26), 1-16
Age of Diagnosis^a^	20.62(11.52), 3-53	-
	n(%)	n(%)
Sex^b^		
Male	78(52)	78(52)
Female	73(48)	73(48)
Location		
England	129(85)	134(89)
Wales	7(5)	5(3)
Scotland	14(9)	10(7)
Northern Ireland	1(1)	2(1)
Additional diagnoses		
ADHD	26(17)	1(1)
Dyslexia	16(11)	4(3)
Dyspraxia	20(13)	1(1)
Dyscalculia	2(1)	0(0)
Intellectual Disability	7(5)	0(0)
Speech/Language Impairment	4(3)	0(0)
Depression	70(46)	23(15)
Anxiety	89(59)	31(21)
Social Anxiety	49(32)	9(6)
Eating Disorders	20(13)	3(2)
Schizophrenia	2(1)	0(0.00)
Bipolar Disorder	4(3)	1(1)
Personality Disorders	10(7)	1(1)
Obsessive Compulsive Disorder	18(12)	1(1)
Post-Traumatic Stress Disorder	7(5)	0(0)
Other	2(1)	0(0)

Note. General cognitive ability was measured using the International Cognitive Ability Resource (Condon & Revelle, 2014). Percentages may not sum to 100 due to rounding

^a^Age of diagnosis information was missing for 4 autistic participants. ^b^15 autistic participants identified as a gender different to their biological sex. It was not possible to match the groups on gender, because no non-autistic participants identified as a gender different to their sex

### Signposting Questionnaire for Autism-2 (SQ-A-2)

The 14-item SQ-A was developed by Jones et al. [41] as a caregiver-report questionnaire for measuring autism signs in children. The items are based on DSM-5 items from the DISCO [42], a clinical interview for caregivers and adults, which facilitates assessment and diagnoses of the autism spectrum in children and adults. These 14 items had previously been identified as the 14 most discriminating interview items in the DISCO DSM-5 algorithm set [43, 44]. In their original form, the items served to guide clinicians as optional prompts within a semi-structured interview, and slight changes were made to adapt them into a questionnaire format while the concept of each item remained intact. For the design of the SQ-A questionnaire, five items were reverse-coded, and a 4-point scale was applied that was combined into a binary scoring scheme using the algorithm syntax applied by Carrington et al. [44].

Across two independent samples of children aged 4–11 years (*N* = 304), the SQ-A has demonstrated good internal consistency (*α* = 0.87), convergent validity with the AQ-10 (*r* =.79) and cross-informant reliability with teacher report (*r* =.60). Jones et al. [41] also showed that the SQ-A differentiated autistic children from non-autistic children, as well as those with other neurodevelopmental conditions (e.g., language impairment), thus demonstrating its specificity to autism.

**Development of the 18-item SQ-A-2** To develop the revised SQ-A-2, we maintained the original wording of the 14-item SQ-A, with self- and informant-report options (only the self-report version is assessed in the current study). For self-report, items were rephrased to reflect first-person, rather than third-person, language. Additionally, we considered inclusion of five additional items from the DISCO. One of these, capturing distress caused by sounds, is one of the most frequently endorsed sensory items in the DISCO [45], and hence was added to better capture sensory sensitivity in the SQ-A-2. The four other items (‘share others’ happiness’; ‘insistence on sameness’; ‘collect objects’; ‘response to visitors’) were chosen as they were highly endorsed (> 30%) by autistic adults and were more common in adults than children [45], and thus may capture behaviours that are particularly relevant to autistic adults. Two other DISCO items (‘talking about repetitive themes’ and ‘repetitive manipulation of objects’), endorsed by > 30% autistic adults in Carrington et al. [45], were considered but ultimately not included in the SQ-A-2, in order to balance clinical utility with brevity.

In line with participatory research practice [46], before the main study, we consulted with four paid autistic adults (two males, two females, aged 22–64) to assess the acceptability and accessibility of the SQ-A-2 items. These consultants did not participate in the main study. In line with their communication preferences, two consulted over email, one over the telephone and one face-to-face. Autistic consultants fed back whether: (i) the items made sense to them, (ii) they found the language acceptable, and (iii) they had any suggested changes. Following this, six experts in autism assessment assessed the consultants’ responses. Four members of this team, made up of three researchers (authors CRGJ, SRL and SJC) and an academic clinical psychologist, suggested adaptations to wording of relevant items. Then, a consultant psychiatrist and mental health nurse, both working in a UK adult autism diagnostic service, reviewed the adaptations suggested by the first four experts. A consensus decision was reached for each item. Crucially, the aim was to balance sensitivity to autistic consultants’ viewpoints with maintaining convergent validity with the original DISCO items. From the 19 items, 15 underwent adaptation in their wording, three remained unchanged, and one item (not in the original 14 ‘signposting set’) was removed entirely. This is because it was considered inappropriate by all autistic consultants (item 19: “I interact appropriately with adults who occasionally visit the house”), and no balance could be found to maintain convergent validity. Whilst the 19-item version was administered in the current study for completeness, no formal analysis was conducted with the 19th item, given its unsuitability. Overall, the development of the SQ-A-2 produced an 18-item Original version, which was the version of the measure prior to consultation, and an 18-item Adapted version that contains 15 items that have undergone adaptation in their wording (see Table 2 for the precise changes in language; and Supplementary Table 1 for additional details on the specific justifications that drove these changes). This Adapted version has a Flesch Reading Ease Score of 62.57, suggesting it is written in ‘Plain English’ and is suitable for 12–14 year olds.

Scoring for the SQ-A-2 follows that of the original SQ-A. Participants rate their agreement with each item on a 4-point Likert scale (‘Definitely Agree’, ‘Slightly Agree’, ‘Slightly Disagree’, ‘Definitely Disagree’) and scoring is binary (1 = endorsing autistic trait; 0 = not endorsing autistic trait), with six reverse scored items. In line with the weighting in the DSM-5 DISCO algorithm, for 10 items, only the most extreme option (i.e., ‘Definitely Agree’ or ‘Definitely Disagree’) is used to endorse an autistic trait, whereas for eight items, endorsing an autistic trait with ‘Slightly Agree’ or ‘Slightly Disagree’ also score 1. Item scores are summed to create a total score ranging from 0 to 18, with greater scores reflecting a greater number of autistic behaviours.

### Autism-spectrum quotient

To assess the convergent validity of the SQ-A-2, participants completed the 10-item AQ-10 [18], the currently recommended brief self-report questionnaire for assessing autism in adults in the UK [24]. Participants rate their (dis)agreement with ten statements capturing autistic traits (e.g., “I find it easy to ‘read between the lines’ when someone is talking to me”; “I usually concentrate more on the whole picture, rather than the small details”), on the same 4-point Likert scale as the SQ-A-2 (‘Definitely Agree’ to ‘Definitely Disagree’), with six reverse scored items. Items are scored in a binary manner, where endorsing an autistic trait (using either Slightly or Definitely) scores 1. Total scores range from 0 to 10, with a greater score reflecting more autistic traits.

### General cognitive ability

All participants had previously completed the International Cognitive Ability Resource (ICAR [47] for a different study [48]. The ICAR is a 16-item online measure of general cognitive ability, with verbal (e.g., verbal reasoning) and non-verbal (e.g., matrix reasoning) elements. It has been well-validated against full-scale IQ tests, including the Wechsler Adult Intelligence Scale [47] and successfully used in both autistic (e.g. [48] and non-autistic (e.g. [49] samples. Total ICAR scores range from 0 to 16, whereby a greater score reflects better general cognitive ability.

### Preference ratings for original and adapted SQ-A-2 items

Autistic participants rated their preference for Original versus Adapted wording of the 15 items that underwent adaptation [1–6, 8–9, 12–18], item-by-item. The three unchanged items [7, 10, 11] were not included. Participants were not made aware of why the items differed or that previous input had been given from autistic consultants.

For each item, participants were presented with the two forms of wording next to each other, labelled A and B. Participants were then asked: “When thinking about your own experiences, which statement do you prefer?”, and used a 5-point Likert scale (‘Strong Preference for A’, ‘Slight Preference for A’, ‘No Preference’, ‘Slight Preference for B’, ‘Strong Preference for B’). Whether A versus B was the Original versus Adapted wording was randomly determined for each item to create two fixed layouts, counter-balanced across autistic participants (Original = A items: 1, 2, 3, 8, 12, 16, 18, or Original = A items: 4, 5, 6, 9, 13, 14, 15, 17). After rating their preference for an item, participants were provided with an optional free-text box to respond qualitatively to the following statement: “If you had a reason for why you preferred one statement over the other, please provide this here”. Quantitative preference ratings were scored from − 2 (strong preference for Original wording) to + 2 (strong preference for Adapted wording), with 0 reflecting no preference. Overall preference scores were calculated by averaging participants’ ratings across all 15 items.

### Procedure

The study received full ethical clearance from Cardiff University’s School of Psychology Research Ethics Committee. Before formal data collection, two paid autistic adults piloted the study, with the aim of improving the accessibility and suitability of the online survey for autistic people. Based on their feedback, appropriate changes were made to the survey. These two consultants did not participate in the main study.

Participants completed the questionnaires online, having been randomly assigned to one of four orders. These orders also included the Repetitive Behaviours Questionnaire-3 [50], which was not relevant to the current study, but is mentioned here for transparency. (1) SQ-A-2 Original, AQ-10, RBQ-3, SQ-A-2 Adapted; (2) SQ-A-2 Original, RBQ-3, AQ-10, SQ-A-2 Adapted; (3) SQ-A-2 Adapted, AQ-10, RBQ-3, SQ-A-2 Original; and (4) SQ-A-2 Adapted, RBQ-3, AQ-10, SQ-A-2 Original. Order assignment did not significantly differ between the Autistic and Non-autistic groups, *χ²* = 0.27, *p* =.97. Next, autistic participants completed preference ratings comparing the Original and Adapted SQ-A-2 items. Finally, all participants reported their age, sex at birth (male, female), gender (male, female, other [e.g., non-binary]), UK country of residence, and whether they had any other diagnosed neurodevelopmental or psychiatric conditions, from a tick-box list (see Table 1). Two attention checks were embedded into the survey, which all participants passed. Participants were debriefed about the study aims and paid in line with *Prolific* guidelines.

### Data analysis

Quantitative data were analysed using JASP version 0.18.1. Multiple forms of the SQ-A-2 were assessed: (i) 14-item SQ-A-2 Original, (ii) 14-item SQ-A-2 Adapted, (iii) 18-item SQ-A-2 Original, and (iv) 18-item SQ-A-2 Adapted. The former two map onto the Original 14-item SQ-A, i.e., DISCO signposting set used in [41], whereas the latter two include the additional DISCO items.

Data were initially inspected for normality of distribution, with the AQ-10 and versions of the SQ-A-2 demonstrating deviation from normal (Shapiro-Wilk test). There were, however, no differences in the pattern of results for non-parametric and parametric analyses (correlations and group wise), so parametric analyses are reported.

**Internal reliability**,** convergent validity and group differences** To assess internal reliability, McDonald’s Omega was used instead of Cronbach’s alpha, as it is more suitable for comparing scales with varying item numbers [51]. A Confirmatory Factor Analysis (CFA) across all participants was conducted to confirm a 1-factor model, whereby SQ-A-2 items load onto one ‘autism’ factor. Ascertaining this would support the use of total SQ-A-2 scores in the current study. Correlational analyses were conducted to investigate convergent validity with the AQ-10, as well as associations between the SQ-A-2 and basic demographics (age, sex, general cognitive ability, and age of autism diagnosis). To compare the Autistic and Non-autistic groups on the AQ-10 and SQ-A-2, independent *t*-tests were conducted. To assess item-specific differences between the groups, the percentage of participants endorsing each item was compared using Chi Squared tests (or equivalent Fisher’s exact tests if *n* < 5 in one or more cells).

**Autistic participants’ wording preferences** One sample Wilcoxon signed-rank tests assessed whether autistic participants’ quantitative preference for Adapted versus Original wording significantly differed from 0 (i.e., no preference). Correlational analyses assessed whether overall preference scores were associated with individual differences within autistic participants (sex, age, general cognitive ability and age of diagnosis).

To explore qualitative reasons for autistic participants’ preferences, free-text responses were analysed using content analysis [52–54]. Content analysis is an effective method to describe and categorise qualitative phenomena across a large dataset, facilitating creation of a systematic list (e.g., as per [55, 56]. Specifically, our content analysis applied a framework with three categories: (i) reasons for preference for Adapted wording, (ii) reasons for preference for Original wording, and (iii) reasons for no preference. After data familiarisation, the first coder (OL) derived the smallest meaning units across the dataset. Meaning units were subsequently collapsed together to form broader codes, and thus a preliminary codebook. Meaning units could only fall under one code (i.e., were not double coded), but two meaning units from one participant could fall under the same code. Two additional coders independently coded the meaning units. All three coders and LAL met on multiple occasions to review the codes, discuss discrepancies, and where appropriate, collapse codes to maintain consistent granularity across the codebook. Finally, LAL checked the codes across the whole dataset against the final codebook. To enable calculation of the percentage of participants endorsing each code, the presence/absence (0/1) of each code was scored per participant. A maximum score of 1 was given per code, regardless of whether participants had multiple meaning units for one code.

## Results

### Internal consistency and convergent validity

All forms of the SQ-A-2 demonstrated a wide range of scores across participants (see Table 3). As would be expected, the range was greater in the Autistic compared to Non-autistic group. All SQ-A-2 scores also demonstrated good-to-excellent internal consistency, comparable with the AQ-10 (ω = 0.83; see Table 3). Overall, the 18-item SQ-A-2 Adapted performed best (ω = 0.85) and the 14-item SQ-A Original least well (ω = 0.78). All SQ-A-2 scores correlated positively and strongly with the AQ-10 (0.71–0.75; see Table 4). Finally, a CFA confirmed good fit for a 1-factor model, suggesting SQ-A-2 items load onto one single ‘autism’ factor (χ²(135) = 305.294, *p* <.001; comparative fit index (CFI) = 0.96; Tucker-Lewis index (TLI) = 0.96; root mean square error of approximation (RMSEA) = 0.07; standardised root mean square residual (SRMR) = 0.13). Although the SRMR was above the recommended threshold of ≤ 0.08, the CFI, TLI and RMSEA all comfortably support a well-fitting 1-factor model [57]. The elevated SRMR likely reflects the highly constrained nature of our 1-factor CFA, particularly in the context of a relatively high number of indicators [58].

**Table 2:** Internal consistency and group comparisons for AQ-10 and SQ-A-2

		All	Autistic		Non-autistic				
	SQ-A-2 Details	ω	M(SD), Med	Range	M(SD), Med	Range	M(SD) Difference,		Group Comparison
AQ-10	–	0.83	7.07(2.12), 8	2–10	2.61(2.02), 2	0–10	4.46(0.10)		*t*(299.20) = 18.70, *p* <.001, *d* = 2.15 [1.87–2.44]
SQ-A-2 Original (14-item)	Original 14 items based on the DISCO ‘signposting set’ (Carrington et al., 2014; 2015) and used in the informant-report SQ-A (Jones et al., 2020).	0.78	5.15(2.27), 5	0–12	1.97(1.94), 1	0–8	3.19(0.33)		*t*(292.88) = 13.10, *p* <.001, *d* = 1.51 [1.25–1.76]
SQ-A-2 Adapted (14-item)	14 items after undergoing adaptation to wording via autistic consultation.	0.81	5.99(2.54), 6	0–14	2.13(1.94), 2	0–8	3.86(0.60)		*t*(280.83) = 14.86, *p* <.001, *d* = 1.71 [1.44–1.98]
SQ-A-2 Original (18-item)	Original 14 items, plus additional DISCO items (item 19 excluded).	0.83	6.72(3.04), 7	0–15	2.25(2.21), 2	0–9	4.47(0.83)		*t*(273.84) = 14.62, *p* <.001, *d* = 1.68 [1.42–1.95]
SQ-A-2 Adapted (18-item)	18 items after undergoing adaptation (item 19 excluded).	0.85	7.70(3.31), 8	0–17	2.48(2.23), 2	0–9	5.22(1.09)		*t*(262.59) = 16.07, *p* <.001, *d* = 1.85 [1.57–2.12]

Note. Internal consistency tests are McDonald’s Omega. Comparisons are Welch’s *t*-tests, and therefore robust to possible non-normality. Values in square brackets are 95% confidence intervals. *d* = Cohen’s *d*

**Table 3:** Correlations between AQ-10, SQ-A-2 and Demographic Variables

	1	2	3	4	5
1 – AQ-10	-	0.71*** [0.65–0.76]	0.72*** [0.67–0.77]	0.73*** [0.67-0.78]	0.75*** [0.70–0.80]
2 – SQ-A-2 Original 14-item	-	-	0.90*** [0.87–0.92]	0.97*** [0.97–0.98]	0.90*** [0.87–0.92]
3 – SQ-A-2 Adapted 14-item	-	-	-	0.89**** [0.86–0.91]	0.98*** [0.97–0.98]
4 – SQ-A-2 Original 18-item	-	-	-	-	0.92*** [0.90–0.93]
5 – SQ-A-2 Adapted 18-item	-	-	-	-	-
6 – Age	− 0.05 [-0.016–0.06]	0.06 [-0.06–0.17]	0.07 [-0.04–0.18]	0.05 [-0.06–0.16]	0.06 [-0.05–0.18]
7 – Sex (0 = Female, 1 = Male)	− 0.01 [-0.12–0.10]	< 0.01 [-0.11–0.12]	0.03 [-0.08–0.59]	− 0.02 [-0.13–0.09]	0.01 [-0.10–0.12]
8 – IQ	0.20*** [0.09–0.31]	0.07 [-0.04–0.18]	0.05 [-0.06–0.17]	0.06 [-0.05–0.17]	0.06 [-0.05–0.17]
9 – Age of Diagnosis^a^	0.22** [0.06–0.37]	0.29*** [0.14–0.43]	0.31*** [0.15–0.45]	0.29*** [0.13–0.43]	0.29*** [0.14–0.43]

Note. Correlations with sex are rank biserial correlations. Values in square brackets are 95% confidence intervals. ^a^Correlations with age of diagnosis are within autistic participants. ***p <.001 **p <.01 *p <.05

### Group differences

Group differences for the different forms of the SQ-A-2 are shown in Table 3. For all SQ-A-2 scores, autistic participants scored significantly greater than non-autistic participants, with large effect sizes demonstrated (*d* = 1.51–1.85). The 18-item Adapted SQ-A-2 showed the largest effect size (*d* = 1.85), which was smaller than that for the AQ-10 (*d* = 2.15), at a marginal level of significance (*z* = -1.95, *p* =.051). When directly comparing the group differences that were found for the Original and Adapted forms, both the 14-item and 18-item Adapted versions demonstrated significantly greater effect sizes than their Original counterparts (14-item, *z* = -2.37, *p* =.020; 18-item, *z* = -2.05, *p* =.040).

Item-by-item between group differences are shown in Table 2. Autistic participants were significantly more likely than non-autistic participants to endorse most Original and Adapted SQ-A-2 items. Accounting for multiple comparisons, significant differences were assumed at *p* <.02 following Holm-Bonferroni correction. Accordingly, the Original form of item 4 (share interests) showed a non-significant group difference (in its Adapted form, a significant group difference was found). Additionally, both the Original and Adapted forms of item 5 (use gestures) showed only marginally significant group differences.

### Correlations with demographic variables

Correlations between the SQ-A-2 and demographic variables are shown in Table 4. Separate correlations within each group are shown in Supplementary Table 2. Across the total sample, SQ-A-2 scores did not significantly correlate with sex, age or general cognitive ability. The same pattern was found for the AQ-10, except for a small positive correlation between AQ-10 scores and general cognitive ability (*r* =.20).

**Table 4:** Item-by-item comparisons of the original and adapted SQ-A-2 Items

Original [O] versus Adapted [A] Items	Count (%) Endorsing Autistic Behaviour		Group Comparison	
	Autistic	Non-autistic	*p*	φ
1. Seek comfort/help when in pain or distress [O] Seek comfort or help when in pain or distress^a^ [A]	68 (45.0) 68 (45.0)	45 (29.8) 45 (29.8)	**0.006** **0.006**	0.16 0.16
2. Do not offer comfort to others [O] Find it difficult to offer comfort if others are upset^b^ [A]	12 (7.9) 39 (25.8)	1 (0.7) 6 (4.0)	**0.003** **< 0.001**	0.18 0.31
3. Avoid other people (move away from them/ have no interest) [O] Tend to avoid others (e.g. move away if I am near them)^b^ [A]	117 (77.5) 124 (82.1)	51 (33.8) 48 (31.8)	**< 0.001** **< 0.001**	0.44 0.51
4. Enjoy sharing many interests with others [O] Enjoy sharing lots of different interests with others^b^ [A]	51 (33.8) 67 (44.4)	37 (24.5) 37 (24.5)	0.08 **< 0.001**	0.10 0.21
5. Use a range of spontaneous gestures that express emotion [O] Use gestures that express emotion^b^ [A]	16 (10.6) 13 (8.6)	5 (3.3) 3 (2.0)	0.01 0.02	0.14 0.15
6. Response to others’ emotions is markedly inappropriate [O] Have difficulty responding to others’ emotions^b^ [A]	10 (6.6) 47 (31.1)	0 (0.0) 2 (1.3)	**0.002** **< 0.001**	0.19 0.40
7. Do not use a pointing gesture to show objects and share interest with others^c^ [O, A]	19 (12.6)	4 (2.65)	**0.002**	0.19
8. Have no interest in friendships or have difficulty in forming friendships [O] Have difficulty with making and keeping friendships^b^ [A]	92 (60.9) 113 (74.8)	38 (25.2) 48 (31.8)	**< 0.001** **< 0.001**	0.36 0.43
9. Spontaneously join in and interact with other people [O] Join in and interact with others without needing to be asked^b^ [A]	119 (78.8) 114 (75.5)	61 (40.4) 38 (25.2)	**< 0.001** **< 0.001**	0.39 0.50
10. Aware of others’ feelings [O, A]	50 (33.1)	7 (4.6)	**< 0.001**	0.36
11. Repeat certain words or phrases out of context, over and over again^c^ [O, A]	43 (28.5)	2 (1.3)	**< 0.001**	0.38
12. Arrange objects in patterns or lines and dislike these to be disturbed [O] Arrange objects in patterns or lines and do not like these to be disturbed^a^ [A]	44 (29.1) 44 (29.1)	1 (0.7) 1 (0.7)	**< 0.001** **< 0.001**	0.40 0.40
13. Have a limited, repetitive pattern of self-chosen activities [O] When left to choose my own activities, choose only a few things that are always the same^b^ [A]	124 (82.1) 135 (89.4)	44 (29.1) 73 (48.3)	**< 0.001** **< 0.001**	0.53 0.44
14. Approaches to others are always one-sided, on own terms [O] Approaches to others can be one-sided^b^ [A]	13 (8.6) 29 (19.2)	1 (0.7) 0 (0.0)	**0.001** **< 0.001**	0.19 0.33
15. Share in other people’s pleasure and happiness as if it were my own [O] Share in others’ happiness as if it were my own^b^ [A]	68 (45.0) 70 (46.4)	34 (22.5) 36 (23.8)	**< 0.001** **< 0.001**	0.24 0.24
16. Insist on things at home remaining the same e.g. furniture staying in the same place, things being kept in certain places or arranged in certain ways [O] Insist on things at home remaining the same (e.g. furniture staying in the same place, things being kept in certain places or arranged in certain ways)^a^ [A]	53 (35.1) 58 (38.4)	5 (3.3) 9 (6.0)	**< 0.001** **< 0.001**	0.40 0.39
17. Collect particular types of objects for no obvious purpose [O] Collect particular types of objects because I want to make a large collection^b^ [A]	38 (25.2) 50 (33.1)	2 (1.3) 5 (3.3)	**< 0.001** **< 0.001**	0.35 0.39
18. Upset by some sounds that do not affect other people e.g. vacuum cleaners, aeroplanes [O] Upset by some sounds that do not affect other people (e.g. vacuum cleaners, aeroplanes)^a^ [A]	77 (51.0) 81 (53.6)	1 (0.7) 4 (2.7)	**< 0.001** **< 0.001**	0.58 0.57

Note. O, Original wording; A, Adapted wording; A > O, Adapted wording endorsed more than Original wording; O > A, Original wording endorsed more than Adapted wording; O = A, no difference in endorsement between Original and Adapted wording

Group comparisons are Chi Squared tests (or equivalent Fisher’s exact tests where *n* < 5 in a cell), comparing groups on endorsement of Original and Adapted items, separately. No group comparisons were made for items 7, 10 and 11 as no adaptations to language were made for these items. Significant differences are assumed at *p* <.02 (boldened), to account for multiple comparisons (Holm-Bonferroni correction). *φ* = Phi effect size. ^a^Very small change to language made (e.g., introduction of parentheses). ^b^more substantial change to language made (e.g., change of meaning of the statement). ^c^No change to language made. ^d^No adapted version of this item

**Autistic group** Within autistic participants, SQ-A-2 scores showed small positive correlations with age (*r*s = 0.19-0.22). SQ-A-2 scores were not associated with general cognitive ability or sex (except for the Original 18-item version, *r* = − .17), whereas AQ scores were associated with having higher general cognitive ability (*r* =.34) and being female (*r* = − .22). Both SQ-A-2 and AQ-10 scores were significantly associated with a later age of diagnosis (*r*s = 0.21-0.31). Given the strong correlation between age and age of diagnosis (*r* =.74, *p* <.001), age of diagnosis correlations were conducted again controlling for age, and a similar pattern of results was found.

**Non-autistic group** Within non-autistic participants, AQ-10 and SQ-A-2 scores showed small, significant associations with being male (*r*s = 0.16-0.21), but not age or general cognitive ability.

### Autistic participants’ wording preferences

Results for autistic participants’ quantitative ratings are shown in Table 5. Accounting for multiple comparisons, significant differences were assumed at *p* <.01 following Holm-Bonferroni correction. Autistic participants generally preferred Adapted versus Original wording. Overall preference ratings, and ratings for 11 out of 15 items, were significantly greater than 0 (where 0 = no preference). Overall preference ratings were significantly associated with having high general cognitive ability (*r* =.30, *p* <.001) and being diagnosed at a later age (*r* =.22 *p* =.008, after controlling for age), but not sex or age (*p*s > 0.13).

**Table 5:** Autistic participants’ quantitative ratings for adapted versus original wording

Item	M(SD), range	Difference from 0		
		*p*	*r*	Direction
1. Seeking comfort	0.24(1.22), -2 to 2	0.025	0.24	A = O
2. Offering comfort to others	1.30(1.09), -2 to 2	**< 0.001**	**0.87**	**A > O**
3. Avoid others	0.75(1.10), -2 to 2	**< 0.001**	**0.71**	**A > O**
4. Share interests	-0.03(1.06), -2 to 2	0.60	− 0.07	A = O
5. Use gestures	0.03(1.19), -2 to 2	0.80	0.03	A = O
6. Respond to others	1.14(1.05), -2 to 2	**< 0.001**	**0.85**	**A > O**
8. Difficulty with friendships	0.45(1.24), -2 to 2	**< 0.001**	**0.43**	**A > O**
9. Interact with others	0.36(0.96), -2 to 2	**< 0.001**	**0.56**	**A > O**
12. Arrange objects	0.37(0.98), -2 to 2	**< 0.001**	**0.54**	**A > O**
13. Self-chosen activities	0.47(1.14), -2 to 2	**< 0.001**	**0.52**	**A > O**
14. One-sided approaches	0.53(1.12), -2 to 2	**< 0.001**	**0.59**	**A > O**
15. Share others’ happiness	-0.12(0.94), -2 to 2	0.10	− 0.22	A = O
16. Insistence on sameness	0.31(0.94), -2 to 2	**< 0.001**	**0.48**	**A > O**
17. Collect objects	0.27(1.18), -2 to 2	**0.01**	**0.31**	**A > O**
18. Upset by sounds	0.36(0.99), -2 to 2	**< 0.001**	**0.51**	**A > O**
Overall	0.43(0.34), -0.40 to 1.47	**< 0.001**	**0.93**	**A > O**

*Note. n* = 147. Autistic participants rated their preference for adapted versus original wording for the 15 items that had undergone wording change on a 5-point scale (-2 = strong preference for original, -1 = slight preference for original, 0 = no preference, 1 = slight preference for adapted, 2 = strong preference for adapted). One-sample Wilcoxon Signed Rank tests assessed whether the average rating for each item significantly differed from 0 (i.e., no preference). Effect sizes are *r* rank biserial correlations. A > 0, Adapted preferred to Original; A = 0, no preference between Adapted and Original. Significant differences are assumed at *p* <.01 (boldened), to account for multiple comparisons (Holm-Bonferroni correction)

Out of 151 autistic participants, 123 gave at least one open-text response to explain their preferences. From these qualitative data, 101 final codes were derived (58 for preference for Adapted wording, 33 for preference for Original wording, and 10 for no preference). Forty-seven of these codes were generic (i.e., shared across items; Supplementary Table 3), whereas 54 codes were item-specific (i.e., specific to a particular item; Supplementary Table 4). Percentages shown in this section reflect the percentage of participants who endorsed a particular code. Overall, a greater percentage of participants endorsed codes related to preferring Adapted wording (codes ranged from 0.8 to 39.0% endorsement) compared to codes related to preferring Original wording (0.8–13.8%) or no preference (1.6–14.6%).

**Generic codes** The most common generic codes for preferring Adapted wording were preferring structure/grammar (35.8%), less negative language (34.1%), better reflecting their personal experiences (32.5%), being easier to understand (28.5%), being less absolute (24.3%), and reading better (22.0%). For Original wording, these were preferring structure/grammar (13.8%), being more specific (13.8%), being easier to understand (12.2%) and providing more choice (12.2%). The most common generic codes for having no preference were neither statement applying (16.2%), and the two statements being too different to compare (14.6%).

**Item-specific codes** Inspecting the most common item-specific codes, three main themes were observed. First, participants preferred Adapted wording because it better explained the difficulty or context underlying their autistic behaviour. For example, 39.0% preferred the Adapted wording of item 2 (offering comfort to others) because it highlights that whilst they want to offer comfort to others, they find it difficult and/or distressing. Similarly, 25.2% preferred Adapted item 6 (respond to others) because it clarifies that their responses may be inappropriate because of a difficulty in responding to others’ emotions. Additionally, 8.1% preferred Adapted item 3 (avoid others) because it allows for the possibility that they avoid people due to a difficulty in socialising.

Second, participants preferred Adapted wording because it better captures a degree of social interest and motivation. For example, 25.2% preferred Adapted item 8 (difficulty with friendships) as it captures that they do have interest in friendships, despite finding them difficult. For item 3 (avoid others), 11.4% participants said they preferred Adapted wording as it highlights that avoiding others is only a tendency, rather than a behaviour they always engage in. Additionally, 8.9% preferred this Adapted item because it reflects their interest in other people.

Third, participants preferred Adapted wording because it better explained their behaviour from their own perspective, rather than an observer’s. For example, 10.6% reported preferring Adapted wording for item 17 (collect objects) because collecting objects does have a purpose to them, even if not obvious to others. Further, 5.7% preferred Adapted item 6 (respond to others) because the Original wording describing their behaviour as ‘inappropriate’ relies solely on an observer’s subjective perspective.

Endorsement for item-specific codes preferring Original over Adapted wording was generally low. The most common code (8.1%) was preferring Original wording for item 17 (collect objects). Typically, this was because participants did not agree with the adapted wording that the purpose of collecting is to make a large collection, instead citing other reasons for collecting.

## Discussion

The current study reports the development of the SQ-A-2; a brief, lifespan autism questionnaire, which has been developed with input from autistic people. Specifically, we tested the SQ-A-2 in its self-report form in a large sample of autistic and non-autistic adults and investigated the impact of adapting language via autistic consultation on item endorsement and measurement properties. The SQ-A-2 showed high internal consistency and construct validity, and demonstrated a large group difference between autistic and non-autistic adults. Further, the Adapted form showed a larger group difference than the Original form, whilst maintaining its measurement properties. Additional quantitative and qualitative data also revealed a clear preference for the Adapted wording amongst autistic adults. Overall, the SQ-A-2, with adapted wording, is acceptable to autistic people and has the potential to be a valid, clinically useful questionnaire for measuring DSM-5 signs of autism. Further work is needed to establish its validity in both self- and informant-report versions across the lifespan.

In developing the SQ-A-2, we employed the 14 signposting DISCO items—as used in the informant-report SQ-A [41]—alongside additional DISCO items to better capture sensory sensitivities, as well as behaviours that might be particularly relevant to adults. Our study therefore enabled the assessment of 14- and 18-item versions of the SQ-A-2, comparing the Original language, as also used in the SQ-A, with Adapted language, as developed in the current study through consultation with autistic people. All versions demonstrated good psychometric properties and large group differences. Further, item-by-item analyses showed that nearly all Adapted items distinguished between autistic and non-autistic participants. The exception was item 5, referring to use of gestures, which showed only a marginally significant group difference. Whilst this item worked effectively in the SQ-A in children [41], it is possible that it is less relevant for capturing autism in autistic adults, especially those diagnosed in adulthood. This is somewhat unsurprising, given growing evidence that autistic people, particularly those without intellectual disability, can develop compensatory strategies and learned behaviours over time, including non-verbal behaviours such as gestures (e.g. [10, 59]. Similarly, Carrington et al. [45], using the same DISCO-based items, found fewer non-verbal communication difficulties in their adult versus child sample. In the current sample, when considering the 14- versus the 18-item Adapted versions, it is noteworthy that the final 18-item Adapted version demonstrated the largest group difference, alongside excellent internal consistency and construct validity, suggesting it is the best performing and therefore the most promising moving forward. Nevertheless, future testing of the SQ-A-2 in its informant-report version and in child samples will be required to establish whether the four items additional to the 14 items work similarly as they do in the current self-report adult sample.

A crucial part of the development of the SQ-A-2 involved consultation with autistic adults to adapt item wording. Surprisingly few autism questionnaires have involved autistic consultation and no studies to our knowledge have assessed the impact of autistic input on item endorsement and measurement properties. Our results suggest that autistic input can play an important role in developing clinically-useful measures. Indeed, SQ-A-2 total scores demonstrated a significantly greater group difference in their Adapted compared to Original form. Accordingly, a greater number of Adapted items reliably differentiated between autistic and non-autistic participants, compared to Original items (e.g., item 4, referring to sharing interests, only showed a significant group difference in its Adapted form). Further, there was a general pattern of larger effect sizes for Adapted compared to Original items. Overall, therefore, the findings highlight the utility of incorporating people’s perspectives and lived experiences into questionnaire development. Our novel approach demonstrates the benefits of carefully balancing peoples’ perspectives with clinical expertise and consideration of clinical criteria, which is rarely done in autism research.

Beyond our investigation of endorsement of SQ-A-2 items, autistic participants overwhelmingly preferred Adapted items to Original ones. Quantitatively, autistic participants significantly preferred Adapted to Original language in most items, and there were no items showing the reverse pattern. Qualitative analysis gave us greater insight into why this was the case, highlighting for example, that the Adapted items: (i) provided more context about the difficulties that underlie atypical behaviour, (ii) better captured social motivation, and (iii) better reflected the autistic person’s perspective rather than an observer’s. These reasons align with the growing literature suggesting that the behaviour and intentions of many autistic people can often be misunderstood by others. For example, despite low social motivation often being associated with autism, many autistic people express a high level of social motivation and desire for social connection [60, 61]. Additionally, these findings follow the wider participatory research movement in autism research, which highlights the benefits of listening to autistic people’s lived experiences and descriptions of their own behaviour, rather than relying solely on the clinician and other observers’ insights [46, 62, 63]. A particular strength of our study is the fact that we engaged a large group of autistic people to give us very specific feedback (i.e., on small changes in language), where much participatory autism research engages a small group of people and/or asks considerably broader questions. Indeed, it is encouraging that in our study over 150 autistic participants endorsed the language adaptations implemented through original discussions with just four autistic consultants.

Our analyses also revealed interesting findings pertaining to how the SQ-A-2 relates to key demographic factors in autistic and non-autistic adults. Here, we focus the discussion on the 18-item Adapted version, as it was the best performing version. First, SQ-A-2 scores were not associated with general cognitive ability across or within participant groups, suggesting that SQ-A-2 scores do not simply reflect differences in intellectual ability. Whereas AQ-10 scores were associated with greater cognitive ability, our results suggest the SQ-A-2 may better capture autistic traits independent of cognitive ability. Second, in terms of age, whilst SQ-A-2 scores were not associated with age across all participants, there was a weak positive correlation with age within autistic participants. Whether this reflects a genuine age effect will be important to investigate in future. Particularly, age was also correlated with age of diagnosis in our autistic group, suggesting awareness of current autistic behaviours may be heightened among our older autistic adults. In line with previous research finding a link between age of diagnosis and autistic traits (e.g. [64], we found that higher SQ-A-2 scores were associated with a later age of diagnosis, even after controlling for current age.

Finally, our findings revealed notable associations with participant sex. In non-autistic participants, SQ-A-2 scores were weakly associated with being male, similar to the AQ-10. This pattern corroborates a substantial body of research showing that autistic characteristics are higher in males than females in the general population (e.g. [65]. No sex differences in the SQ-A-2 were found within autistic participants, in line with large-scale previous research (e.g., [66]. This is likely indicative of the fact that all autistic participants, male or female, have reached the clinical threshold for diagnosis. That male autistic participants were not more likely to endorse SQ-A-2 items than females is promising, given widespread concerns that current autism measures are biased towards males [67, 68]. This stands in contrast to the AQ-10, which showed significantly higher scores for autistic females than autistic males in the current study. Ultimately, further psychometric testing to establish whether the SQ-A-2 measures the same latent construct in males and females will be critical.

The findings in the current study converge to indicate the utility of the SQ-A-2 as a brief and accessible self-report measure of autistic traits in intellectually able adults. The measure provides autistic people, family members and professionals insight into the pattern of a person’s autistic behaviours across 18 of the most discriminating items from a clinical interview, the DISCO [3, 4]. In its current form, and in line with dimensional and needs-based approaches to neurodivergence [69], the measure could be used to help identify areas where someone may need support, regardless of whether they have received an autism diagnosis. This could be done in a clinical context but also has scope for self-directed use, where a person’s profile of answers could direct them to relevant support and resources. Further, the pattern of responses could be used to support clinical decision-making about whether a diagnostic referral would be useful. Similarly, the SQ-A-2 may be a useful and brief way to help a person decide whether they should pursue a clinical diagnosis. In the future, and following further psychometric evaluation, the SQ-A-2 has the potential to serve as a robust and time-saving tool that positively impacts the lengthy and complex process of DSM-5 autism diagnosis. For example, future analysis of the sensitivity and specificity of the SQ-A-2 in a prospective study with participants referred from frontline services could provide a reliable cut-off to inform referral decisions. Finally, for both future research and clinical support, it would also be beneficial to apply the 1–4 point scale instead of, or in addition to, the reduced binary scoring system. This would provide additional variation in pattern for individuals and may potentially enhance the power of future psychometric testing [70].

### Limitations

Our findings should be interpreted in light of some limitations. First, whilst our large sample of adults were well-matched (e.g., on age, sex and general cognitive ability), given the self-report nature of the study, autistic people with above average cognitive ability are likely to have been over-represented. As such, our findings may be less relevant to autistic people with an additional intellectual disability, who made up only 5% of the Autistic group in the current study, and are poorly represented in autism research more generally [71]. To address this, future testing of the informant-report version of the SQ-A-2, which can be completed by a caregiver or friend, will be essential. More generally, preference for Adapted versus Original wording was also associated with greater general cognitive ability, which suggests that people with lower IQ may have different or fewer preferences with regards to language. Ensuring that all autistic people’s views on language are represented therefore remains a critical priority for autism research.

Second, the SQ-A-2 reflects the majority of existing autism questionnaires in being developed in a Western high-income country, with the linguistic preferences of our autistic participants likely reflecting their wider cultural context as well as their autistic identity. Indeed, previous research has shown that adapting autism screening instruments for use in low- and middle-income countries has often resulted in poorly performing instruments, likely reflecting underlying cultural and socioeconomic factors that influence question interpretation [72]. Even within high-income countries, racial and ethnic minorities are underrepresented in autism research [73, 74], reflecting a pervasive issue with cultural and contextual biases. We did not collect ethnicity data, so we cannot currently speak to how ethnically heterogenous our sample was and therefore how relevant the findings may be to an ethnically diverse range of autistic people. More generally, in line with calls for open, replicable science in autism research (e.g., [75], it will be important to assess whether the current findings replicate in larger, more diverse samples (e.g., in terms of language and intellectual ability and ethnicity).

Third, whilst the original SQ-A was successfully shown to differentiate autistic children from those with other neurodevelopmental conditions [41], we were unable to assess the discriminant validity of the SQ-A-2 in the current adult sample. As such, it will be important to establish that the SQ-A-2 distinguishes autistic people from people with a related neurodevelopmental (e.g., ADHD) or psychiatric (e.g., Social Anxiety Disorder) condition. This consideration is particularly pertinent given the high co-occurrence of autism with many other neurodevelopmental and psychiatric conditions. For example, it is estimated that approximately 28% of autistic people have ADHD, 20% have an anxiety disorder, 12% have disruptive, impulse-control or conduct disorders, and 11% have a depressive disorder [76]. Testing the specificity of the measure in this way will be critical if it is to be useful in the earliest stages of the diagnostic process; for example, for referring people from primary care services to specialist services for a full diagnostic assessment. Fourth, whilst there is evidence that the online platform *Prolific* enables high-quality data collection (e.g. [77] and enables recruitment of participants who might not otherwise be able to attend in-person testing (e.g., due to financial and health reasons), it was not possible to clinically verify autistic participants’ diagnoses. Future investigation of the SQ-A-2 Adult in in-person research, including clinical research via diagnostic services, is now necessary.

Finally and most critically, it will now be necessary to assess the full psychometric properties of the SQ-A-2, including the self- and informant-report versions, in large samples of autistic and non-autistic adults and children. The scope of analyses within the current study was limited due to insufficient statistical power. Full factor analytic investigation, for example, will enable us to refine the measure further, if necessary, and ascertain sub-scales, where appropriate. For example, it could be explored whether there are separate factors that align with the two main DSM 5 diagnostic categories. Additioanally, measurement invariance analyses, which ascertain whether a tool is measuring the same latent structure across groups, will establish if the SQ-A-2 captures the same ‘concept’ of autism across, for example, males and females, children and adults, intellectual ability and disability, and autistic and non-autistic people. Assessing this in relation to sex is particularly pertinent, given that the AQ, in its different versions, has been shown to measure a different latent construct in males and females (e.g. [68, 78]. Finally, receiver operating characteristic (ROC) analysis may be useful when assessing the sensitivity and specificity of the measure, especially when informing its use in a clinical context.

## Conclusions

The current study developed a revised lifespan questionnaire for measuring autism signs, the SQ-A-2. The SQ-A-2 may be particularly advantageous over other brief autism questionnaires as it is closely aligned with current DSM-5 criteria, is applicable for both children and adults, and has been developed with autistic input. Our findings revealed that in adults, the SQ-A-2 demonstrates robust internal consistency and construct validity, and shows large group differences between well-matched groups of autistic and non-autistic people. Promisingly, SQ-A-2 scores do not appear to be associated with general cognitive ability or sex within autistic people. Further, our novel approach of testing the impact of adapting questionnaire wording via consultation with autistic people has highlighted the benefit of carefully balancing autistic people’s language preferences with clinical criteria during the measurement development process. Future research is now required, including full psychometric evaluation of the SQ-A-2—in both its self- and informant-report forms—in samples of autistic children and adults. Overall, the SQ-A-2, with adapted wording, holds promise as a questionnaire measure of autism signs, which we hope will serve both important clinical and research purposes at a time when demand for diagnostic assessments for autism are higher than ever.

## Supplementary Information

Below is the link to the electronic supplementary material.

Supplementary Material 1

## Data Availability

The anonymised quantitative data are available from the corresponding author(s) on reasonable request.

## Abbreviations

ASD: Autism Spectrum Disorder
AQ-10: 10-Item Autism–Spectrum Quotient
SQ-A: Signposting Questionnaire for Autism (Child)
SQ-A-2: Revised Signposting Questionnaire for Autism (Lifespan)
DISCO: Diagnostic Interview for Social and Communication Disorders
NICE: National Institute for Health and Care Excellence
ICAR: International Cognitive Ability Resource
RAADS–14: 14–item Ritvo Autism Asperger Diagnostic Scale
RBQ-3: Repetitive Behaviours Questionnaire
