# Supplementary material for: The Signposting Questionnaire for Autism-2 (SQ-A-2): using input from autistic adults to inform questionnaire development
Source: Mol Autism. 2026 Apr 15;17:22. doi: 10.1186/s13229-026-00714-3 (PMC13151257; doi:10.1186/s13229-026-00714-3)
Supplement: Supplementary file 1 — Supplementary Material 1 [file 13229_2026_714_MOESM1_ESM.docx]

**Supplementary Materials**

Supplementary Table 1

Comparison of Original and Adapted Wording with Justification for Change

| Item | Original wording | Adapted wording | Justification for changes* |
| --- | --- | --- | --- |
| 1 | Seek comfort/help when in pain or distress | Seek comfort or help when in pain or distress | Autistic consultants generally felt this be fine, minor adaptation made (‘or’) for readability. |
| 2 | Do not offer comfort to others | Find it difficult to offer comfort if others are upset | Three of the autistic consultants raised issues. The item was considered blunt. It was also felt to rest on an assumption that the person knows how to offer comfort. It was mentioned that a person may try to offer comfort but not know how, or offer comfort but do the wrong thing. Adjusted to reflect perceived difficulties in offering comfort. |
| 3 | Avoid other people (move away from them/ have no interest) | Tend to avoid others (e.g. move away if I am near them) | Autistic consultants generally thought this was fine but issues with clarity were discussed. Adjusted to improve clarity. |
| 4 | Enjoy sharing many interests with others | Enjoy sharing lots of different interests with others | The use of ‘many’ caused confusion for two autistic consultants, and one reflected that words without clear quantification can be difficult. ‘Lots’ was considered a better word. |
| 5 | Use a range of spontaneous gestures that express emotion | Use gestures that express emotion | This raised issues for all autistic consultants. Particularly, ‘spontaneous’ was not defined, and answering requires insight. It was not possible to agree wording that relied on other people’s feedback (e.g. ‘people tell me…’) as this would introduce additional issues. However, ‘spontaneous’ and ‘range’ were removed to reduce demands on insight. |
| 6 | Response to others’ emotions is markedly inappropriate | Have difficulty responding to others' emotions | All autistic consultants raised issues with this item, with one describing it as ‘insensitive’ and another as ‘offensive’. Two had difficulties in understanding ‘markedly’, whilst another thought it contributed to the insensitivity of the question. The item was adjusted to focus on the difficulty a person might experience, whilst removing comment on the quality of responses. |
| 7 | Do not use a pointing gesture to show objects and share interest with others |  | Autistic consultants generally thought this was fine. No amendments made. |
| 8 | Have no interest in friendships or have difficulty in forming friendships | Have difficulty with making and keeping friendships | One autistic consultant said that the two parts to the item made it hard to answer, whilst another suggested that *keeping* friendships was an important dimension not currently captured. Adjustments were made to include the keeping of friendships and to narrow the focus of the question. |
| 9 | Spontaneously join in and interact with other people | Join in and interact with others without needing to be asked | Autistic consultants generally thought this was fine but did suggestions about improving clarity (e.g. use of ‘spontaneous’). Reworded to improve clarity. |
| 10 | Aware of others’ feelings |  | Autistic consultants generally thought this was fine. No amendments made. |
| 11 | Repeat certain words or phrases  out of context, over and over again |  | Autistic consultants generally thought this was fine, although comments about needing insight were made. No amendments made. |
| 12 | Arrange objects in patterns or lines and dislike these to be disturbed | Arrange objects in patterns or lines and do not like these to be disturbed | Autistic consultants generally thought this was fine. Some suggestions were made about clarity and a minor adjustment was made (‘do not like’). |
| 13 | Have a limited, repetitive pattern of self-chosen activities | When left to choose my own activities, choose only a few things that are always the same | Autistic consultants shared some concerns about this item, including that the wording felt ‘blunt’ and ‘medical’, and that ‘limited’ is a subjective term. Amendments included softening the language and reducing the subjectivity. |
| 14 | Approaches to others are always one-sided, on own terms | Approaches to others can be one-sided | Three autistic consultants raised issues. One did not like the potential assumption that the person does not care for others (this felt ‘nasty’), whilst another thought ‘always’ was too absolute. Removed ‘are always’ and ‘on own terms’ to reflect this feedback. |
| 15 | Share in other people’s pleasure and happiness as if it were my own | Share in others’ happiness as if it were my own | Three of the autistic consultants had comments, they were all different but related to how the question could be interpreted. The word ‘pleasure’ was considered to complicate the item and was removed. |
| 16 | Insist on things at home remaining the same e.g. furniture staying in the same place, things being kept in certain places or arranged in certain ways | Insist on things at home remaining the same (e.g. furniture staying in the same place, things being kept in certain places or arranged in certain ways) | Autistic consultants generally liked this question and thought the examples were helpful. The examples were put in brackets to improve clarity. |
| 17 | Collect particular types of objects for no obvious purpose | Collect particular types of objects because I want to make a large collection | Two of the autistic consultants found the word ‘purpose’ difficult in the context of the question, particularly as purpose is subjective. Amended to remove the comment about purpose. |
| 18 | Upset by some sounds that do not affect other people e.g. vacuum cleaners, aeroplanes | Upset by some sounds that do not affect other people (e.g. vacuum cleaners, aeroplanes) | Autistic consultants generally thought this was fine. No amendments made. |

*Note this is brief summary of the justification, focusing on the main reasons for the adjustments that were made. It does not capture all views, nor the complex iterative discussions that led to the final amended versions of the items.

Supplementary Table 2

Correlations between AQ-10, SQ-A-2 and Demographic Variables by Group

|  | 1 | 2 | 3 | 4 | 5 | 6 | 7 | 8 |
| --- | --- | --- | --- | --- | --- | --- | --- | --- |
| 1 – AQ-10 | - | .49*** [.35–.60] | 0.50*** [.37–.61] | .50*** [.37–.61] | .54*** [.41–.64] | .03 [-.13–.19] | -0.22** [-.36– -.06] | .34*** [.19–.47] |
| 2 – SQ-A Adult Original 14-item | .49*** [.36–.60] | - | .82*** [.76–.87] | .95*** [.93–.96] | .82*** [.76–.87] | .20* [.04–.35] | -.11 [-.26–.06] | .12 [-.04–.28] |
| 3 – SQ-A Adult Adapted 14-item | .45*** [.32–.57] | .86*** [.81–.90] | - | .79*** [.72–.84] | .96*** [.94–.97] | .22** [.06–.37] | -.07 [-.23–.09] | .08 [-.08–.24] |
| 4 – SQ-A Adult Original 18-item | .49*** [.36–.60] | .98*** [.97–.99] | .84*** [.79–.88] | - | .84*** [.79–.88] | .19* [.03–.34] | -.17* [-.32– -.01] | .07 [-.09–.23] |
| 5 – SQ-A Adult Adapted 18-item | .47*** [.34–.59] | .86*** [.81–.90] | .98*** [.97–.98] | .87*** [.83–.91] | - | .21** [.05–.36] | -.12 [-.28–.04] | .08 [-.08–.24] |
| 6 – Age | -.05 [-.21–.11] | .03 [-.13–.19] | .07 [-.10–.22] | .03 [-.13–.19] | .06 [-.10–.22] | - | - | - |
| 7 – Sex (0 = Female, 1 = Male) | .19* [.04–.34] | .13 [-.03–.29] | .18* [.03–.33] | .16* [<.01–.32] | .21** [.06–.36] | - | - | - |
| 8 – IQ | .11 [-.05–.26] | -.06 [-.21–.11] | -.07 [-.23–.09] | -.03 [-.19–.13] | -.05 [-.21–.11] | - | - | - |

*Note.* Correlations within the Autistic group are shown above the diagonal and correlations within the Non-autistic group are shown below the diagonal. Correlations with sex are biserial rank correlations. Values in square brackets are 95% confidence intervals. ****p* < .001 ***p* < .01 **p* < .05

Supplementary Table 3

Generic Qualitative Codes for 1) Preference for Adapted Wording, 2) Preference for Original Wording, and 3) No Preference.

|  | | Preference for Adapted | | Preference for Original | | | No Preference | |
| --- | --- | --- | --- | --- | --- | --- | --- | --- |
| Code | Example quotes | Item | % | Item | % | Item | | % |
| Prefer structure/grammar | “*phrased better*”; “*prefer with brackets*” | 1-4, 6, 12-14, 16, 18 | 35.8 | 1, 4-5, 9, 12-14, 16, 18 | 13.8 | - | |  |
| Less negative | “*Original sounds cruel*”; “*seems warmer*” | 3, 5-6, 8, 13-14, 17 | 34.1 | 12, 15 | 2.4 | - | |  |
| Better reflects personal experience | “*Describes what I would do*”; “*more accurate for me*” | 1-6, 8-9, 12-15, 17 | 32.5 | 1, 3-5, 9, 12-14, 17 | 10.6 | - | |  |
| Easier to understand | “*clearer*”; “*simpler phrase*” | 1-6, 8-9, 13-14, 16-18 | 28.5 | 1-6, 14-17 | 12.2 | - | |  |
| Less absolute/definite | “*better describes the nuance*”; “*more flexible*” | 1-3, 6, 8, 14 | 24.4 | 5 | 0.8 | - | |  |
| Reads better | “*better flow*”; “*more natural*” | 1-4, 9, 12-13, 16, 18 | 22.0 | 1, 4-5, 8, 13-16 | 8.1 | - | |  |
| Adapted & Original are too different to compare | “*difference between the two [statements] is drastic”; “'no interest’ and 'difficulty’ are different”* | 3, 5-6, 8, 14, 17 | 16.3 | 5-6, 14, 17 | 2.4 | 2-3, 5-6, 8-9, 12, 14, 17 | | 14.6 |
| More specific | “*more detailed explanation*”; “*less vague*” | 1-2, 4, 8-9, 13, 17 | 11.4 | 4-6, 8, 14-15 | 13.8 | - | |  |
| Provides more choice | “*covers more scenarios*”; “*allows for a wider remit of behaviour to be covered*” | 1, 3, 5, 9, 16 | 8.9 | 1, 8, 15 | 12.2 | - | |  |
| Prefer aesthetics | “*looks better*”; “*paratheses make it neater*” | 1, 12, 16, 18 | 8.9 | 1 | 0.8 |  | |  |
| More concise | “*less wordy*”; “*more succinct*” | 2, 4-5, 14-15 | 6.5 | 1-2, 4, 12-13 | 10.6 | - | |  |
| Less clinical | “*Original reads like a clinical manual*”; “*Adapted is less unnecessarily clinical*” | 9, 13 | 6.5 | - |  | - | |  |
| Perspective from self, rather than observer | “*Original is an outsider’s perspective*”; “*prefer personal assessment of self versus by others*” | 6, 13, 17 | 4.9 | - |  | - | |  |
| Original reinforces autism stereotypes | “*'no interest in friendships’ feeds into emotionless robots narrative*”; “*Original reinforces stereotypes about autistic people having no empathy*” | 2, 5, 8 | 4.9 | - |  | - | |  |
| Requires less self-insight | “*Adapted is more helpful as a person might not realise their responses are inappropriate*”; “*some may know why they collect items and other may not*” | 6 | 4.1 | - |  | 16-17 | | 1.6 |
| Suggested change to wording | “*would prefer ‘and/or’*”; “*use ‘usually’ instead of ‘always’*" | 1, 9, 17 | 2.4 | - |  | 14-16, 18 | | 2.4 |
| Adapted & Original are too similar to compare | “*cannot tell the difference between the two statements*”; “*phrases are similar*” | 2, 9 | 2.4 | - |  | 1-2, 4-6, 9, 12-16, 18 | | 11.4 |
| Neither apply | “*Neither are particularly appropriate to me*”; “*don’t like either*” | 14, 17 | 2.4 | - |  | 3-6, 8-9, 12-18 | | 16.3 |
| Both unclear | “*both are imprecise*”; “*what is meant by an ‘approach’ is ambiguous*” | 3, 14 | 1.6 | 5 | 4.1 | 1, 4, 9, 14 | | 6.5 |
| Depends on context | “*whether they offer comfort depends on previous relationship with person*”; “*gestures vary by culture and environment*” | 2 | 1.6 | - |  | 5 | | 1.6 |
| May not apply to everyone | “*this won't work for other autistic people*”; “*some people may collect items for reasons other than making a large collection*” | 13, 17 | 1.6 | - |  | - | |  |
| Not sure | “*not sure why*”; “*not entirely sure why I prefer Adapted*” | 9, 12 | 1.6 | 14, 15, 17 | 3.3 | - | |  |
| Better differentiates autistic from non-autistic people | “*Adapted could refer too much to non-autistic people too*”; “*not specific enough to help with diagnosis*” | - |  | 5, 13, 14 | 3.3 | - | |  |
| More expressive | “*Original is more expressive of emotion*” | - |  | 12 | 0.8 | - | |  |
| Both true | “*relate to both*”; “*both can be true for me*” | - |  | - |  | 6, 9, 13 | | 2.4 |
| Indifferent | “*don’t mind*”; “*both look fine*” | - |  | - |  | 4, 16-17 | | 1.6 |
| Dislike both | “*don’t like the wording of either statement*”; “*dislike both statements*” | - |  | - |  | 8, 16 | | 1.6 |

*Note.* Generic codes (i.e., shared across items) generated from autistic participants’ open-ended responses. Each generic code for Preference for Adapted, Preference for Original and No Preference is represented alongside example quotes from participants and the associated item numbers. % reflects the percentage of autistic participants (*n* = 123) who endorsed each particular code. Items 7, 10 and 11 were not included in the autistic participants’ preference ratings as these items did not undergo adaptation in their wording.

Supplementary Table 4

Item-specific Qualitative Codes for 1) Preference for Adapted Wording, and 2) Preference for Original Wording.

| Adapted [A] Wording | Code | Example quotes | % | Original [O] Wording | Code | Example quotes | % |
| --- | --- | --- | --- | --- | --- | --- | --- |
| **1) I seek comfort or help when in pain or distress** | Comfort and help are different things | “*comforting and helping are two different things…slash implies two words function the same*” | 14.6 | **1) I seek comfort/help when in pain or distress** | Comfort and help are the same | “*comfort and help are similar things*” | 1.6 |
| **2) I find it difficult to offer comfort if others are upset** | Want to offer comfort to others but it is difficult/distressing | “*desire to offer comfort but have difficulty*” | 39.0 | **2) I do not offer comfort to others** | Do not offer comfort to others | “*Don’t know how to do it so rather would not offer*” | 1.6 |
|  | Not offering comfort is not lack of caring | “*it is not due to not caring*”; “*[O] suggests lack of empathy*” | 5.7 |  | Avoid others | “*prefer to avoid people*” | 0.8 |
|  | Put in effort | “*I at least try to offer comfort*”; “*[O] sounds like they don't want to try*” | 4.1 |  | - |  |  |
|  | Mismatch with other people’s expectations for offering comfort | “*prefer to offer practical solutions to a problem rather than empty words*”; “*people are not comfortable with pragmatic solutions*” | 2.4 |  | - |  |  |
| **3) I tend to avoid others (e.g. move away if I am near them)** | Tendency to avoid others, not always | “*always want to [avoid people] but can't always do it*”; “*don't avoid people depending on my mood*” | 11.4 | **3) I avoid other people (move away from them/ have no interest)** | Avoid others | “*safer to stay away from people*” | 4.1 |
|  | Do have interest in others | “*have interest in other people*”; “*find people fascinating*” | 8.9 |  | - |  |  |
|  | Avoid others because of difficulty | “*[A] highlights struggle to socialise*”; “*Don’t lack interest, just about comfort*” | 8.1 |  | - |  |  |
|  | Avoid others because of preference for space | “*like to keep physical distance*”; “*I* *have a bubble and do not like others being closer than that*” | 6.5 |  | - |  |  |
| **4) I enjoy sharing lots of different interests with others** | Have lots of *different* interests | “*Have wide range of interests and can switch between them*”; “*lots of different interests denotes the variability/breadth of my interests*” | 3.3 | **4) I enjoy sharing many interests with others** | Do not have lots of interests | “*I only have a few interests*”; “*have interests but not ‘lots’*” | 2.4 |
| **5) I use gestures that express emotions** | Gestures are not spontaneous | “*gestures are never spontaneous*”; “*spontaneous is at odds with most autistic people's learned behaviours*” | 8.1 | **5) I use a range of spontaneous gestures that express emotion** | Gestures are not spontaneous | “*I can perform gestures learned for specific emotions but they are not authentic*”; “*they don't come naturally”* | 6.5 |
|  | Do not use a *range* of gestures | “*use gestures but not sure of their range*”; “*use the same gestures”* | 2.4 | - | Do use spontaneous gestures | “*my gestures are more often spontaneous*”; “*masked for so long, no longer realise I’m doing it*” | 6.5 |
| **6) I have difficulty responding to others' emotions** | Difficulty with responding to others’ emotions | “*have difficulty knowing what to say*”; “*[O] implies doing something rude on purpose rather than having difficulty*” | 25.2 | **6) My response to others’ emotions is markedly inappropriate** | Knows responses are inappropriate | “*most of the time my response is one I understand is not correct*” | 1.6 |
|  | Responses are not *markedly* inappropriate | “*[A] is not knowing how to respond to a widow while [O] is bursting into awkward laughter and asking them on a date*”; “*not inappropriate but not the norm*” | 5.7 |  | Adapted implies giving no response | “*[A] implies no response whatsoever*” | 1.6 |
|  | ‘Inappropriate’ is subjective | “*inappropriate from whose perspective?*”; “*inappropriate suggests there is a universal set of rules for emotions and reactions…there aren't*” | 5.7 |  | - |  |  |
|  | Atypical communication is not their fault | “*[O] implies doing something rude on purpose*”; “*not my fault for communicating wrong*” | 5.7 |  | - |  |  |
|  | ‘Inappropriate’ is incorrect | “*autism is not inappropriate*”; “*sometimes I do respond appropriately to others' emotions*” | 4.1 |  | - |  |  |
|  | Put effort into responding appropriately | “*[A] acknowledges the effort it takes to respond appropriately*”; “*[O] does not account for amount of effort put in*” | 1.6 |  | - |  |  |
| **8) I have difficulty with making and keeping friendships** | Want friendships but they are difficult | “*would love to have friends but no idea how to make friends*”; “*have some interest in friendships but not always easy*” | 25.2 | **8) I have no interest in friendships or have difficulty in forming friendships** | Not interested in friendships | “*no interest in making friends*”; “*not interested in friendship*” | 3.3 |
|  | Specific difficulty in keeping friendships | “*easy to make friends but difficult to keep them*”; “*difficulty with maintaining friendships in initial stages*” | 7.3 |  | Some other autistic people not interested in friendships | “*some autistic people aren't interested in making friends*”; “*reflects autistic friends who seem not to care”* | 1.6 |
|  | Do have interest in friendships | “*I like having friends*”; “*have an interest in forming friendships*” | 3.3 |  |  |  |  |
| **9) I join in and interact with others without needing to be asked** | Do need invitation | “*needing to be asked is a big part”*; “*not spontaneous...more likely to join if it feels like it is expected*” | 7.3 | **9) I spontaneously join in and interact with other people** | Likes spontaneously | “*likes spontaneously*”; “*likes the word spontaneously*” | 1.6 |
|  | Original implies joining in without invitation | “*[Original] implies they randomly intrude on a group*”; “*joining without an invitation is rude*” | 4.1 |  | ‘Being asked’ is too restrictive | “*being asked excludes other ways people try to engage them*”; “*If they join in it's because they want to*” | 1.6 |
|  | Do not need invitation | “*can be involved without prompting*”; “*mainly joins in when not asked*” | 2.4 |  | - |  |  |
| **12) I arrange objects in patterns or lines and do not like these to be disturbed** | Stronger language | “*do not like seems stronger than 'dislike'*”; “*stronger language*” | 2.4 | **12) I arrange objects in patterns or lines and dislike these to be disturbed** | - |  |  |
|  | Less strong language | “*dislike is too strong*” | 0.8 |  | - |  |  |
| **13) When left to choose my own activities, choose only a few things that are always the same** | Activities are not repetitive | “*limited number of activities I enjoy but not always done in the same way and intensity*”; “*some activities done at the same time each day but others not done regularly*” | 4.9 | **13) I have a limited, repetitive pattern of self-chosen activities** | - |  |  |
|  | Activities are a choice | “*hobbies are out of choice not out of compulsion*” “*[A] feels like they have more control”* | 3.3 |  | - |  |  |
|  | Activities are not limited | “*don’t feel like I limit my interests*”; “*[activities] are not limited*” | 2.4 |  | - |  |  |
| **14) Approaches to others can be one-sided** | Approaches are not always one-sided | “*always is too strong, depends on the situation*”*;* “*not always one-sided depending on mood*” | 10.6 | **14) My approaches to others are always one-sided, on own terms** | Approaches are one-sided/on own terms | “*[approaches] can be one-sided*”; “*On my own terms is the important part*” | 2.4 |
|  | Approaches are not intentionally one-sided | “*tries hard to avoid this but sometimes can be one-sided”;* “*[O] is selfish…[A] can just be thoughtless*” | 5.7 |  | Have to make the effort with others | “*I have to do all the work*”; “*I try to fit in by guessing and adapting myself to the other person's terms*” | 1.6 |
|  | Approaches are on others’ terms | “*don’t socialise on own terms…automatically mask when meeting a new person*” | 0.8 |  | - |  |  |
| **15) Share in others' happiness as if it were my own** | Dislikes ‘pleasure’ | “*pleasure sounds inappropriate*”; “*dislike the word pleasure*” | 4.1 | **15) I share in other people’s pleasure and happiness as if it were my own** | Likes ‘pleasure’ | “*prefer the word pleasure added*” | 0.8 |
|  | Happiness and pleasure are different things | “*Pleasure and happiness are two different things…ask about pleasure in a different question*”; “*limiting to either happiness or pleasure makes more sense*” | 1.6 |  | - |  |  |
| **17) Collect particular types of objects because I want to make a large collection** | Like to have a large collection | “*Collecting makes me happy so would not stop at a small collection*”; “*collect because want a lot of them*” | 5.7 | **17) I collect particular types of objects for no obvious purpose** | Reasons to collect, other than for a large collection | “*does not collect to have a large collection…collects because they like things*”; “*size collection does not matter”* | 8.1 |
|  | Collecting has a purpose | “*collecting always has a purpose*”; “*purpose may not be obvious to others but it is to me*” | 10.6 |  | Collecting has no purpose | “*no purpose to it*”; “*collects things for the sake of it*” | 2.4 |

*Note.* Item-specific codes (i.e., specific to a particular item) generated from autistic participants’ open-ended responses. Each item-specific code is represented alongside example quotes from participants. % reflects the percentage of autistic participants (*n* = 123) who endorsed each particular code. There were no item-specific codes generated for No Preference and/or item 18. Items 7, 10 and 11 were not included in the autistic participants’ preference ratings as these items did not undergo adaptation in their wording.
